# P-1324. Evaluation of Sulbactam-Durlobactam Activity and Synergy Against Highly Drug-Resistant Acinetobacter baumannii, Including an MBL-Producing Strain

**DOI:** 10.1093/ofid/ofaf695.1512

**Published:** 2026-01-11

**Authors:** Justin Halim, Jeannete Bouzo, Valerie J Carabetta

**Affiliations:** Cooper Medical School of Rowan University, Ocean, NewJersey; Rowan-Virtua School of Osteopathic Medicine, Camden, NewJersey; Cooper Medical School of Rowan University, Ocean, NewJersey

## Abstract

**Background:**

*Acinetobacter baumannii* is a multidrug-resistant pathogen of urgent public health concern. Sulbactam-durlobactam (SUL-DUR), a newly approved β-lactam/β-lactamase inhibitor combination, is highly effective against carbapenem-resistant *A. baumannii* (CRAB), but resistance via metallo-β-lactamases (MBLs) and PBP3 mutations has emerged. Optimizing therapy through synergistic antibiotic combinations may preserve SUL-DUR activity.

**Table 1.**
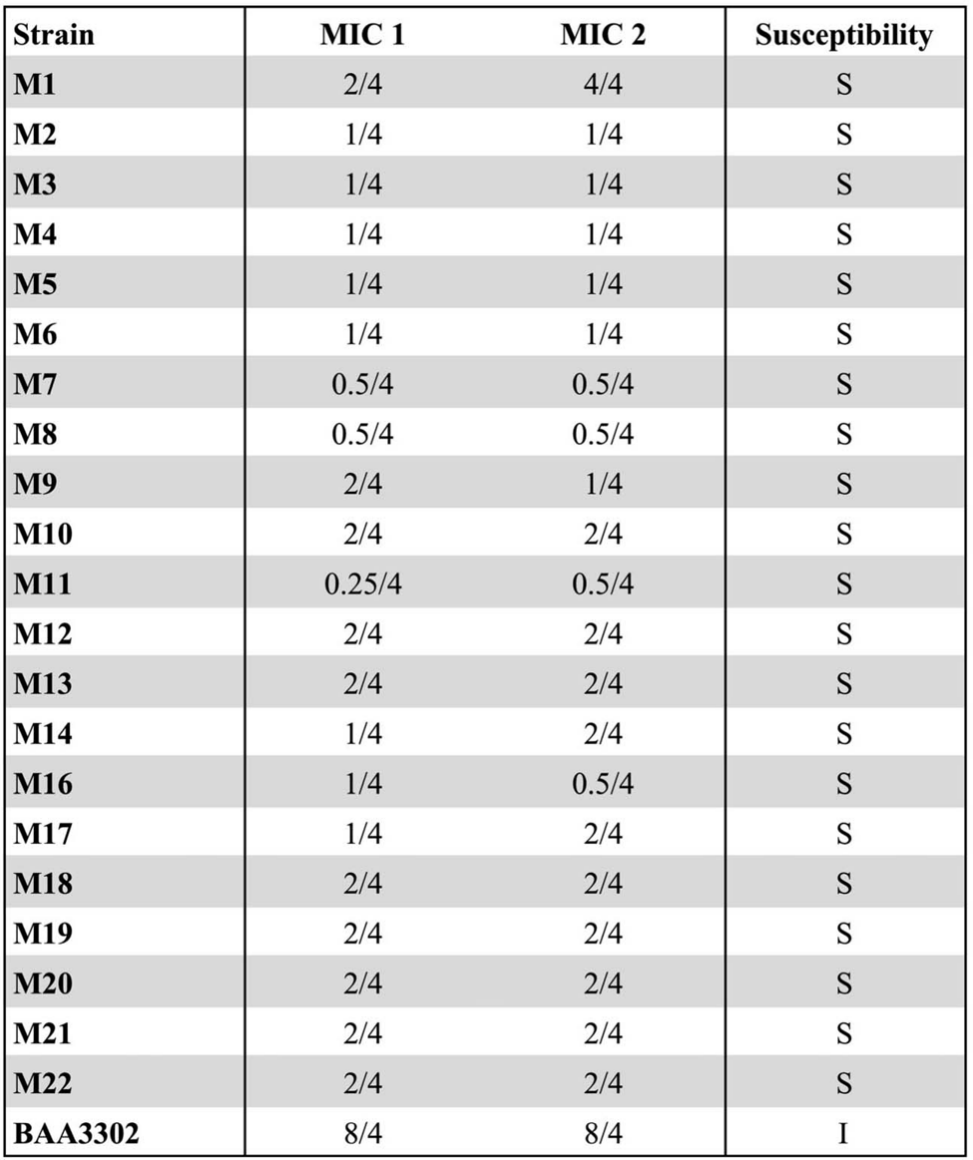
Antibiotic susceptibilities of each A. baumannii isolate to SUL-DUR Strains from our collection are labeled M1-M22, and an additional MBL-harboring strain called BAA-3302 is included. Note that strain M15 was excluded from the study, as it was not A. baumannii. MIC values were determined at least two independent times, with both values presented. Strains that are susceptible are denoted as S; strains with intermediate susceptibility are denoted as I; strains that are resistant are denoted as R, according to CLSI standards.

**Table 2.**
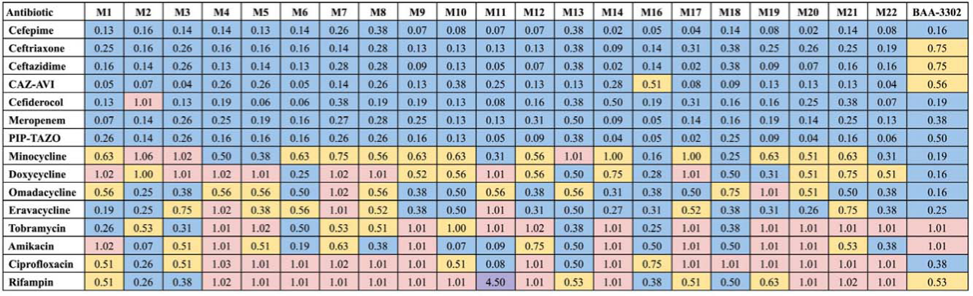
Fractional inhibitory concentration index (FICI) values obtained from SUL-DUR in combination with various antibiotics against A. baumannii strains FICI values in the synergistic range (≤0.5) are reported in blue; FICI values in the additive range (0.5-1.0) are reported in yellow; FICI values indicating no interaction (1.0-4.0) are reported in pink; FICI values in the antagonistic range (>4.0) are reported in purple.

**Methods:**

We evaluated the *in vitro* activity of SUL-DUR alone and in combination with 15 antibiotics against 22 *A. baumannii* strains. M1-M22 are XDR/PDR clinical isolates and BAA-3302 is an MBL-producing strain. MICs were determined by broth microdilution. Checkerboard assays assessed synergy, with FICI values calculated. Select combinations were validated with static time-kill assays (TKAs) against strains M1 and BAA-3302.

**Table 3.**
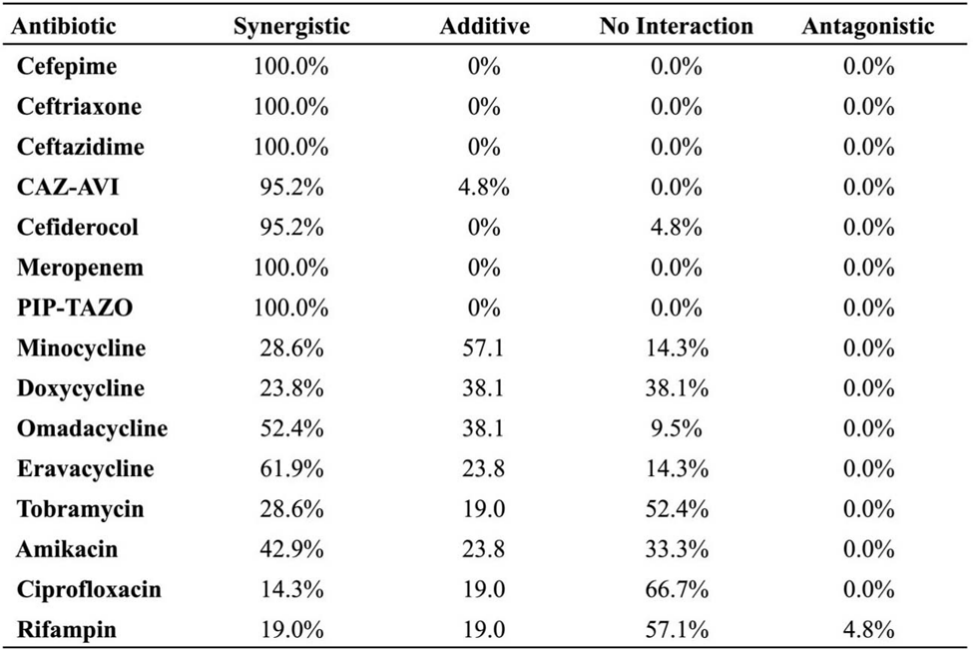
Rates of interactions between SUL-DUR and various antibiotics Rates of synergistic, additive, indifferent, and antagonistic effects between antibiotics paired with SUL-DUR against strains M1-M22. Note strain BAA-3302 was excluded from this analysis.

**Figure 1.**
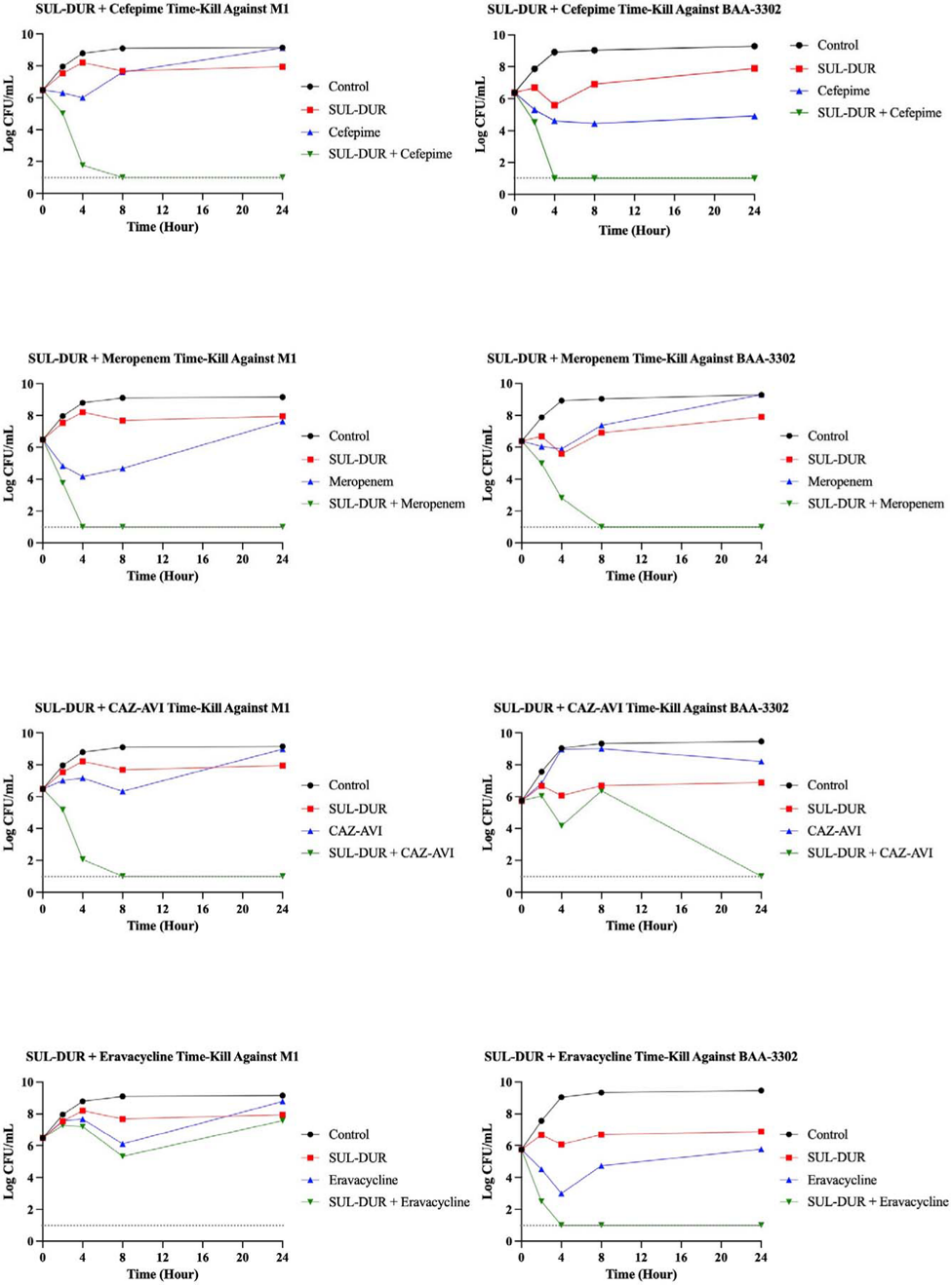
Time-Kill Analysis of SUL-DUR Combinations Against A. baumannii Strains M1 and BAA-3302 Time-kill assays evaluating the bactericidal activity of SUL-DUR in combination with cefepime, meropenem, CAZ-AVI, or eravacycline against two clinical isolates of A. baumannii: M1 (left panels) and BAA-3302 (MBL-carrier; right panels). Four parallel conditions were assessed for each combination: (1) untreated growth control, (2) SUL-DUR alone, (3) comparator antibiotic alone, and (4) SUL-DUR combined with the comparator antibiotic. Bacterial counts (log_10_ CFU/mL) were measured at 0, 2, 4, 8, and 24 hours. SUL-DUR combinations demonstrated rapid and sustained synergistic bactericidal activity (≥2-log_10_ CFU/mL reduction compared to the single most active agent) in both strains. Only eravacycline did not demonstrate synergism with SUL-DUR against M1 (bottom left). The dashed line indicates the lower limit of detection.

**Results:**

SUL-DUR demonstrated potent activity (MIC ≤4/4) against 21/22 strains; the MBL-producing strain BAA-3302 showed intermediate susceptibility (MIC 8/4) (Table 1). Whole-genome sequencing of select strains revealed PBP3 mutations and diverse β-lactamase genes, including *blaOXA* and *blaADC* variants, while BAA-3302 harbored *blaNDM-1*. Checkerboard assays showed β-lactams—including cefepime, meropenem, and ceftazidime-avibactam (CAZ-AVI)—were synergistic with SUL-DUR against >95% of strains (Tables 2 and 3). Eravacycline and omadacycline demonstrated modest synergy. In BAA-3302, synergy was retained with cefepime, meropenem, cefiderocol, and all tetracyclines. TKAs confirmed bactericidal synergy for cefepime, meropenem, and CAZ-AVI, with ≥2 log₁₀ killing at 24 hours, against both strains M1 and BAA-3302 (Figure 1).

**Conclusion:**

SUL-DUR demonstrates broad *in vitro* efficacy against XDR/PDR A. baumannii, including when combined with β-lactams and select tetracyclines. Synergistic combinations with cefepime, meropenem, or CAZ-AVI may enhance treatment efficacy and suppress resistance, even against MBL-producing strains.

**Disclosures:**

All Authors: No reported disclosures

